# Exploring *Aeromonas dhakensis* in Aldabra giant tortoises: a debut report and genetic characterization

**DOI:** 10.1186/s12866-024-03203-w

**Published:** 2024-03-07

**Authors:** Chenxu Zhao, Panpan Qin, Shuai Li, Zilu Chen, Tianliang Wang, Qunchao Liang, Weishi He, Zeyu Peng, Yurong Yang, Zhifeng Peng, Yongtao Li

**Affiliations:** 1 College of Veterinary Medicine, Henan Agricultural University, Zhengzhou, 450046 China; 2 Henan Yinji Jiabao Amusement Park Management Co. LTD, Zhengzhou, 452376 China; 3 Zhengzhou Central Hospital Affiliated to Zhengzhou University, Zhengzhou, 450001 China; 4 College of Veterinary Medicine, Henan University of Animal Husbandry and Economy, Zhengzhou, 450046 China

**Keywords:** Aldabra giant tortoise, *Aeromonas dhakensis*, Multidrug resistance, Pathogenicity, Public health

## Abstract

*Aeromonas dhakensis (A. dhakensis)* is becoming an emerging pathogen worldwide, with an increasingly significant role in animals and human health. It is a ubiquitous bacteria found in terrestrial and aquatic milieus. However, there have been few reports of reptile infections. In this study, a bacterial strain isolated from a dead Aldabra giant tortoise was identified as *A. dhakensis* HN-1 through clinical observation, matrix-assisted laser desorption ionization–time of flight mass spectrometry (MALDI-TOF/MS), and gene sequencing analysis. Subsequently, to evaluate its pathogenicity, the detection of virulence genes and mice infection experiments were performed. *A. dhakensis* HN-1 was found to contain seven virulence genes, including *alt*, *ela*, *lip*, *act*, *aerA*, *fla*, and *hlyA*. Mice infected with *A. dhakensis* HN-1 exhibited hemorrhage of varying degrees in multiple organs. The half-maximal lethal dose (LD_50_) value of *A. dhakensis* HN-1 for mice was estimated to be 2.05 × 10^7^ colony forming units (CFU)/mL. The antimicrobial susceptibility test revealed that *A. dhakensis* HN-1 was resistant to amoxicillin, penicillin, ampicillin and erythromycin. This is the first report of *A. dhakensis* in Aldabra giant tortoises, expanding the currently known host spectrum. Our findings emphasize the need for One Health surveillance and extensive research to reduce the spread of A. dhakensis across the environment, humans, and animals.

**Supplementary Information:**

The online version contains supplementary material available at 10.1186/s12866-024-03203-w.

## Introduction

Over the past 20 years, the world has experienced various zoonotic disease outbreaks, with a growing number of emerging zoonoses being discovered in human or animal populations, owing to the application of innovative diagnostic technologies. Zoonotic diseases, or zoonoses, encompass bacterial, parasitic, viral, and fungal infections that can be transmitted from wild or domesticated animals to humans [1]. The genus Aeromonas is widely distributed in diverse ecological systems, including underground water, drinking water, river water, oceanic water, irrigating water, and regenerating wastewater [2–7]. Some species of Aeromonas are opportunistic pathogenic bacteria that infect a wide range of animals such as fish, amphibians, aquatic animals, and reptiles, as well as humans [8–15]. The most common Aeromonas infections include enter gastritis, skin and soft-tissue infections, bacteremia, septicemia, as well as other infections affecting the hepatobiliary system, respiratory system, bones, and joints [9, 10, 15, 16]. These bacteria can spread through contaminated water or food, and exposure to fecal pollution, posing a zoonotic challenge to animals and humans [17]. The high risk of Aeromonas spillover and burden has been heightened by extensive interactions among animals, humans, and ecosystems. Thus, a comprehensive One Health approach, involving multisectoral collaboration, is strongly warranted to address this complex health threat [18].

*A. dhakensis* (formerly *A. aquariorum*), which was previously misidentified as *A. hydrophila*, was first isolated from children with diarrhea in Dhaka [19]. It has also been found in skin tissue of ornamental fish in Portugal [7, 20], diseased eels [7] and diseased dolphin in Spain [14]. Furthermore, it has been detected in diseased fish in South Korea [21] and Mexico [22], striped catfish in Vietnam [15], a diseased sea lion [16] and farmed Siamese crocodiles in China [23]. *A. dhakensis* has caused serious infections in humans [10, 24–26], demonstrating higher virulence compared to other *Aeromonas* species [23, 27]. In fact, it is considered the most infectious species within the *Aeromonas* genus [23, 24, 27]. Because of its rising occurrence in animals and humans worldwide, *A. dhakensis* is now recognized as an important emerging pathogen in the realm of zoonotic infections.

The Aldabra giant tortoise (*Aldabrachelys gigantea*) is one of the largest vertebrate tortoises in the world. It is also known for its extraordinarily long lifespan. As one of the two remaining species of giant tortoise on earth, the Aldabra giant tortoise has become increasingly popular in zoos worldwide. Recently, researchers have discovered that the lineage-specific genome of the Aldabra giant tortoise is related to disease development, which may explain its extended lifespan [28]. However, there is a lack of available data on diseases caused by causative agents in Aldabra giant tortoises [29]. Therefore, it is crucial to investigate zoonotic pathogens that may affect the health of these endangered tortoises and pose potential risks to public health. In this study, a fatal infection in an Aldabra giant tortoise in China was described, with the causative agent identified as *A. dhakensis* for the first time. Furthermore, the possible route of transmission, antimicrobial resistance, and the significance of *A. dhakensis* on public health are also discussed.

## Materials and methods

### Case report and pathological examination

**Fig. 1:**
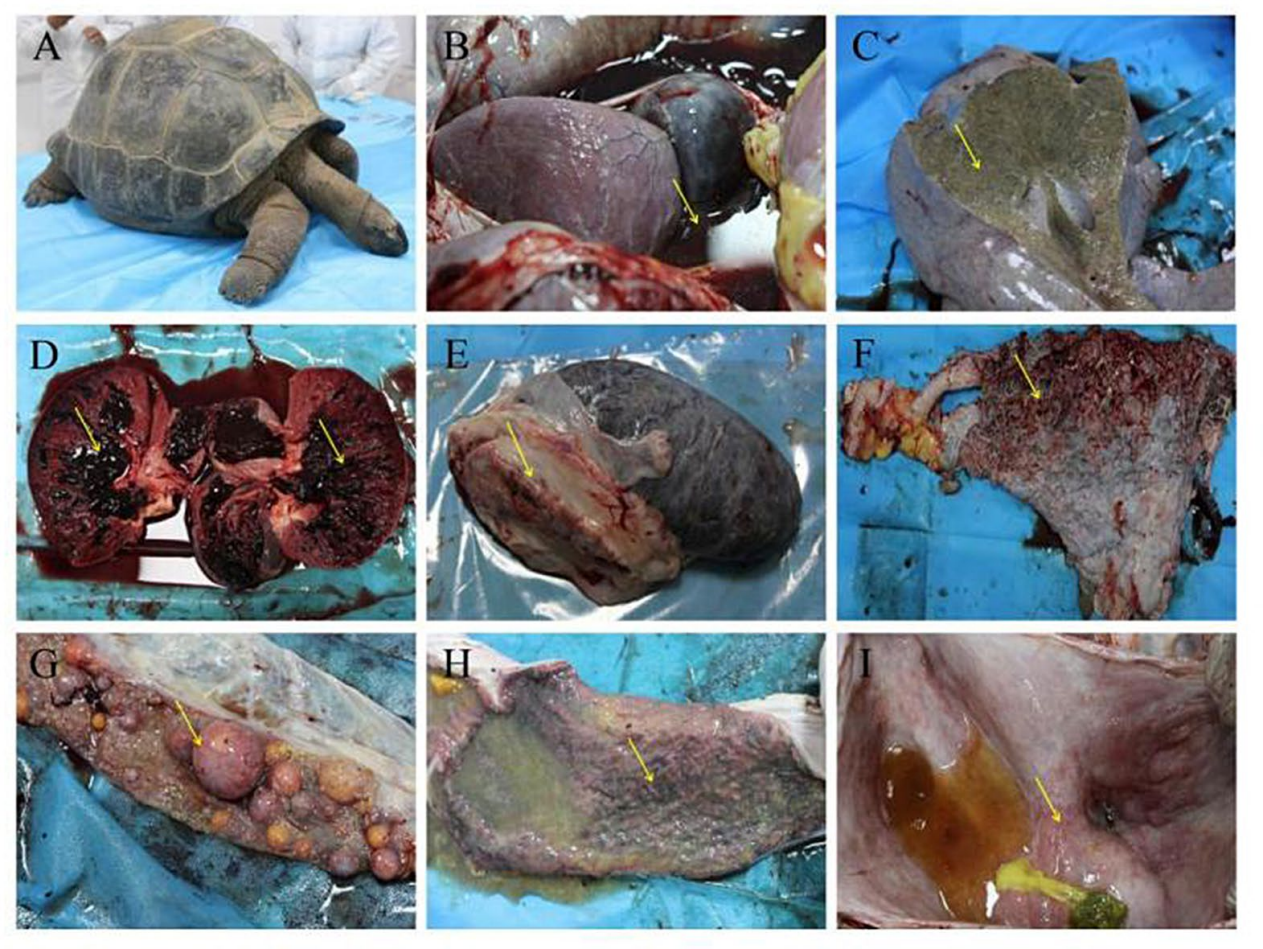
Gross lesions of the dead Aldabra giant tortoise in Henan, China. (**A**) Effusion pleural effusion. (**B**) Fibrinous exudation and hemorrhage in heart. (**C**) Swelling in the greyish-yellow liver. (**D**) Hemorrhage with multiple papillae in kidneys. (**E**) Edema and necrosis in spleen. (**F**) Congestion in lung. (**G**) Hemorrhage on the surface of the follicle. (**H**) duodenal ulcer with necrotic foci in cecum. (**I**) hemorrhage in bladder

In August 2022, a 47-year old female Aldabra giant tortoise at an animal zoo in Zhengzhou, Henan province, China, died spontaneously. The tortoise, weighing 125 kg and measuring 110 cm in body length, showed no appetite and appeared listless until its death. While there were no visible wounds or obvious symptoms on the body surface, the fresh corpse was subsequently transported to the Veterinary Diagnostic Laboratory at Henan Agricultural University for further examination. Following disinfection of the tortoise’s surface with 75% ethanol, a postmortem examination was carried out to conduct a pathological examination of various organs. Subsequently, samples were aseptically collected from the internal organs, including the liver, kidney, heart, lung, and spleen, using sterile scalpels, scissors, and sterile inoculating loops for histopathological and laboratory examinations (Fig. 1A).

### Isolation, morphological and MALDI-TOF MS detection of bacteria

All samples were streaked on various media, including blood agar plates with 5% erythrocytes (Biocell, Zhengzhou, China), Trypticase soy agar (TSA) plates with 5% sheep blood (AOBOX, Beijing, China), brain–heart infusion (BHI) plates with 5% sheep blood (AOBOX, Beijing, China), MacConkey (MAC) plates (AOBOX, Beijing, China). All the plates were incubated in 37 °C incubator for 24 h. The separate cultured colonies were subjected to bacteriological culture, Gram staining, and microscopy. Subsequently, further species identification was performed with MALDI-TOF MS (Bruker, USA) according to the manufacturer’s instructions [30].

### Molecular identification

The genomic DNA from pure culture colonies was extracted using a genomic DNA purification kit (Tiangen Biotech, Beijing, China) and stored at − 20 °C until used as PCR templates. Further molecular identification was performed using sequencing and phylogenetic analysis of the 16S *rRNA* and housekeeping gene *gyrB*. The 16S *rRNA* amplification was performed using universal primers 27 F (5ʹ-AGAGTTTGATCCTGGCTCAG‐3ʹ) and 1492R (5ʹ‐GGCTACCTTGTTACGACTT‐3ʹ) and PCR protocols as described previously [31]. The universal primers for amplification of *gyrB* genes were gyrB-F (5ʹ -GAAGTCATC ATGACCGTTCTGCAYGCNGGNGGNAARTTYGA‐3ʹ) and gyrB-R (5ʹ- AGCAGGGTACGGATGTGCGAGCCRTCNACRTCNGCRTCNGTCAT‐3ʹ), the *gyrB* genes were amplified using PCR protocols as described previously [32]. The 16S *rRNA* and *gyrB* gene sequences were determined by Sangon Biotech (Shanghai, China). Then we determine the closest matching sequences in GenBank by the BLAST search algorithm (National Center for Biotechnology Information, NCBI). Based on 16S *rRNA* gene and *gyrB* gene sequences, phylogenetic evolutionary trees were constructed and analyzed using MEGA 6.0 software by the neighbor-joining method with 1000 bootstrap replicates, respectively.

### Virulence gene detection

Total DNA of the newly identified *Aeromonas* isolate was extracted with a Bacterial Genomic DNA Isolation Kit (Sangon Biotech, Shanghai, China) and were used as PCR temples for subsequent virulence gene detection. Eight virulence associated genes were selected as virulence marker, including *aerA* (aerolysin), *hlyA* (hemolysin), *fla* (flagella), *alt* (heat-labile cytotonic enterotoxin), *ast* (heat-stable cytotonic enterotoxin), *act* (cytotoxic enterotoxin), *lip* (lipase) and *ela* (elastase). The primers used to detect virulence genes were listed in Table S1. The PCR products were collected by electrophoresis in 1.0% agarose gel with DL2000 DNA marker (Takara, Shanghai, China).

### Mice infection experiments

Thirty-six 3-week-old Kunming mice weighing 18–22 g were obtained from Henan Experimental Animal Center to confirm the pathogenicity of the newly identified strain. Before the experiment initiation, all mice were housed in a pathogen-free environment with a 12-hour light-dark cycle for 7 days. There were five groups of mice, each consisting of six mice, which were inoculated intraperitoneally with the newly identified strain at different doses: 6.6 × 10^7^, 2.2 × 10^7^, 7.3 × 10^6^, 2.4 × 10^6^ and 8.15 × 10^5^ CFU/mouse (0.3 mL/mouse). The control group, on the other hand, was inoculated with 0.3 mL of sterile PBS per mouse. For 7 days, the mortality and symptoms of the mice were recorded on a daily basis. To determine the LD_50_ value, the Reed and Muench method was used [33]. Bacteriological detection and pathological examination were performed on three randomly selected dead mice. The soft tissues were fixed with 10% neutral-buffered formalin for more than 24 h, manually sectioned with a microtome into 4 μm slices, and then stained with hematoxylin and eosin for light microscopy. Additionally, the hearts, livers, and spleens of the dead mice were used for re-isolation and identification of the bacteria [34]. At indicated time points, control mice were euthanized by anesthetic deepening (300 mg/mL of ketamine and 30 mg/mL of xylazine).

### Antimicrobial susceptibility

The antimicrobial susceptibility was measured with the Kirby-Bauer disk diffusion method according to Clinical Laboratory Standards Institute document (CLSI, 2020). Briefly, the cultured bacterial colonies were diluted with normal saline equal to 0.5 McFarland standard and 100 µL of bacteria was spread on Mueller–Hinton agar medium plates (AOBOX, Beijing, China), and then antibiotic discs (Hangzhou Tianhe Microbiological Reagent, Hangzhou, China) were placed at least 30 mm apart on the plate. After incubation at 37 °C for 24 h, the diameter of the inhibition zone of inhibition around each disk was measured and recorded. The following antibiotics classified into nine different categories were tested, aminoglycosides: gentamicin (10 µg), amikacin (30 µg), spectinomycin (100 µg), kanamycin (30 µg); quinolones: norfloxacin (10 µg), levofloxacin (5 µg), ciprofloxacin (5 µg); tetracyclines: doxycycline (30 µg); sulfonamides: sulfamethoxazole (1.25 µg); β-lactams: amoxicillin (20 µg), penicillin (10 U), ampicillin (10 µg), ceftriaxone (30 µg), cefoperazone (75 µg), ceftazidime (30 µg); macrolides: erythromycin (15 µg); fosfomycin: fosfomycin (200 µg); carbapenems: meropenem (10 µg) and rifampicin: rifampicin (5 µg). The *Escherichia coli* ATCC 25,922 was used as a quality control strain for antibiotic susceptibility testing.

## Results

### The dead Aldabra giant tortoise exhibited acute lesions in multiple organs

After necropsy, the main symptoms of the diseased Aldabra giant tortoise were lethargy. Based on hemorrhage in multiple organs, we preliminarily diagnosed that the Aldabra giant tortoise died from septicemia. The necropsy findings revealed lesions in various organs, including effusion pleural effusion and hemorrhage in the heart (Fig. 1B), swelling in the greyish-yellow liver (Fig. 1C), hemorrhage with multiple papillae in the kidneys (Fig. 1D), edema and necrosis in the spleen (Fig. 1E), congestion in the lung (Fig. 1F), hemorrhage on the surface of the follicle (Fig. 1G), duodenal ulcer with necrotic foci in the cecum (Fig. 1H), and hemorrhage in the bladder (Fig. 1I).

**Fig. 2:**
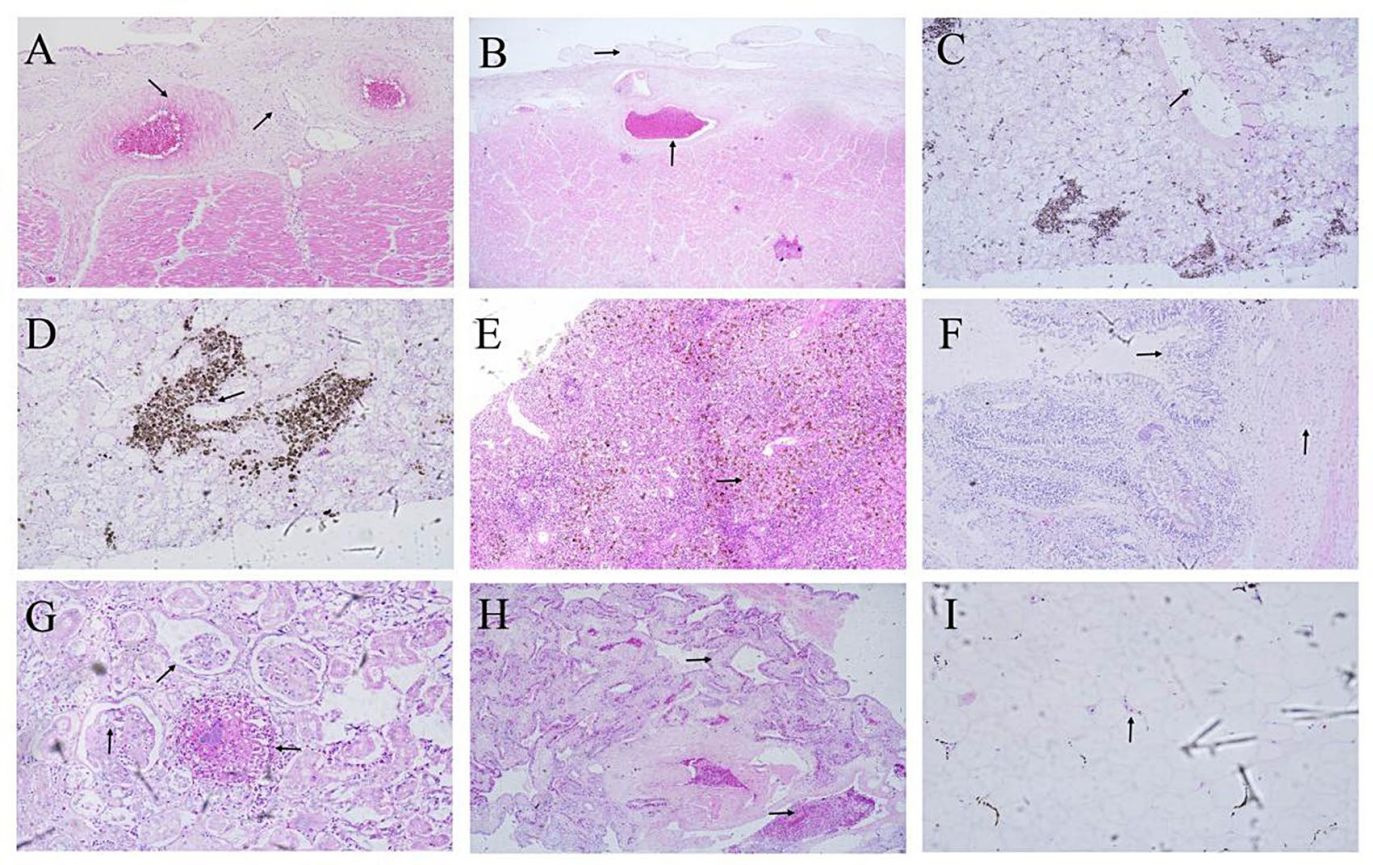
Histological lesions of the dead Aldabra giant tortoise. HE. (**A**) Vessel walls hyperplasia and thickened with infiltration of heterophile leukocytes (200×). (**B**) Verrucous hyperplasia in the epicardium. hyperemia and protein exudation in the blood vessel (100×). (**C**) Fatty degeneration and vacuolar degeneration of hepatocytes, and fused into large vacuoles in the live (400×). (**D**) Interstitial collagen fibroplasia with infiltration of a great quantity of heterophil leukocyte in the liver (400×). (**E**) Spleen trabecular loosing, White pulp necrosis, a great quantity of scattered golden clumps or particles in the spleen (100×). (**F**) Intestinal villus fusion, villous epithelial cell degeneration and necrosis. A large number of neutrophils infiltrated in the intestinal lamina propria (200×). (**G**) Infiltration of a great quantity of neutrophils around the purulent foci. Fibrin exudation in the renal capsule cavity. Necrosis of renal tubular epithelia in the kidney (400×). (**H**) Infiltration of red blood cells and protein-like exudates in the alveoli pulmonum matrix and alveolar cavity (100×). (**I**) Fatty degeneration in the bladder (400×)

The histological examination of the dead Aldabra giant tortoise showed major pathological changes, including extensive hemorrhage, vacuolar degeneration, necrosis, and infiltration of inflammatory cells in multiple organs and tissues. Infiltration of a great quantity of heterophil leukocytes was observed in the fibrotic vascular wall (Fig. 2A), along with collagen fibers hyperplasia and discontinuous verrucous hyperplasia in the epicardium (Fig. 2B). Hepatocytes showed fatty degeneration and vacuolar degeneration, forming large vacuoles (Fig. 2C), while the liver exhibited interstitial collagen fibers proliferation with infiltration of a large number of heterophilic leukocytes (Fig. 2D). The spleen showed white pulp necrosis and scattered golden particles (Fig. 2E). In the intestines, there was villous epithelial cell degeneration, fusion of intestinal villi, multiple focal suppurative lesions in the lamina propria and submucosa, and infiltration of a large number of neutrophils in the lamina propria (Fig. 2F). The renal tissue exhibited multiple purulent lesions with infiltration of neutrophils, glomerular atrophy, hemorrhage, renal capsule cavity enlargement, fibrin exudation, and degeneration and necrosis of renal tubular epithelial cells (Fig. 2G). Alveolar interstitial widening, a significant amount of inflammatory cell infiltration, and the presence of red blood cells and protein-like exudates in the alveolar space and interstitial were observed (Fig. 2H). Additionally, bladder steatosis was observed (Fig. 2I).

### The strain HN-1 belongs to the genus *Aeromonas*

**Fig. 3:**
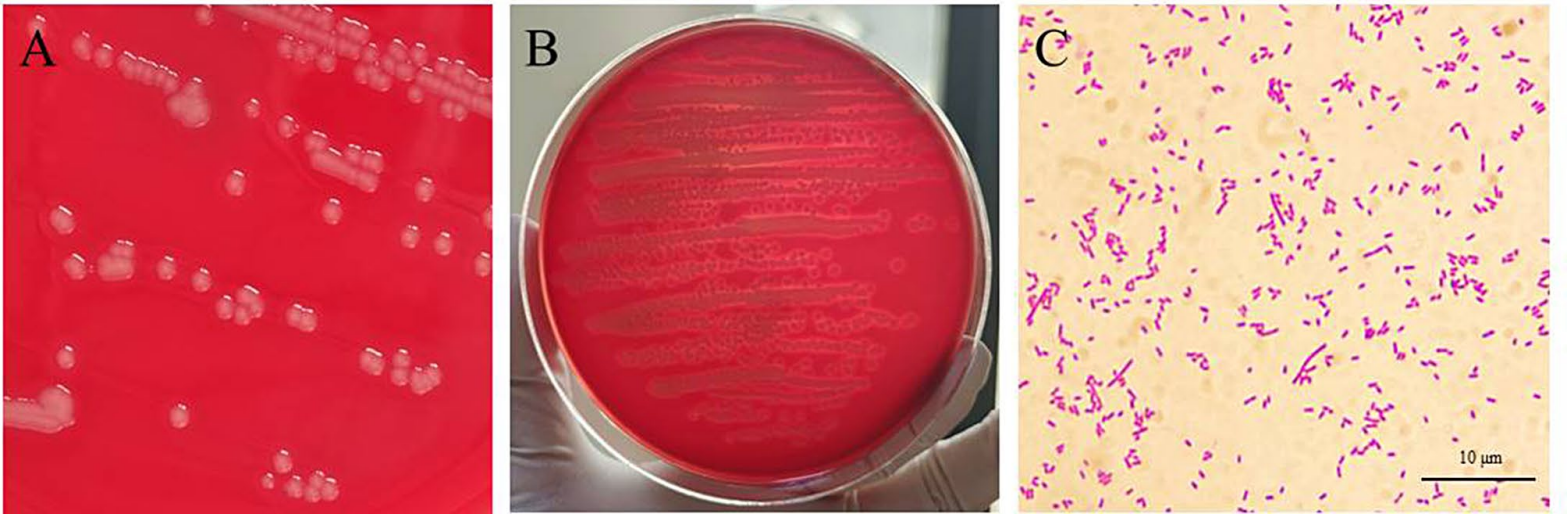
Colony morphology and microscopic findings of bacterial isolate HN-1. (**A**, **B**) The bacteria grew well and produced a β-hemolytic zone on sheep blood agar plates. (**C**) Gram-negative bacilli were observed under an oil immersion lens, with different sizes and staining depths.

The tissues collected aseptically were inoculated onto TSA plates, MAC plates, BHI plates and sheep blood plates for bacterial isolation and culture. The results indicated that the same bacteria, termed as HN-1, were cultured on plates inoculated with the heart blood, pericardial effusion, spleen, lung, kidney, and liver tissue. The colony of HN-1 was 2 millimeters in diameter, round, smooth, moist and pale grey (Fig. 3A), and beta-hemolysis was observed on sheep blood plates (Fig. 3B). The newly isolated bacterial strain HN-1 was Gram-negative, short rod with blunt ends, arranged in single or double (Fig. 3C). In MALDI-TOF MS, HN-1 is best matched with *Aeromonas hydrophila* with a score value of 1.855.

### The strain HN-1 was identified as *A. dhakensis*

**Fig. 4:**
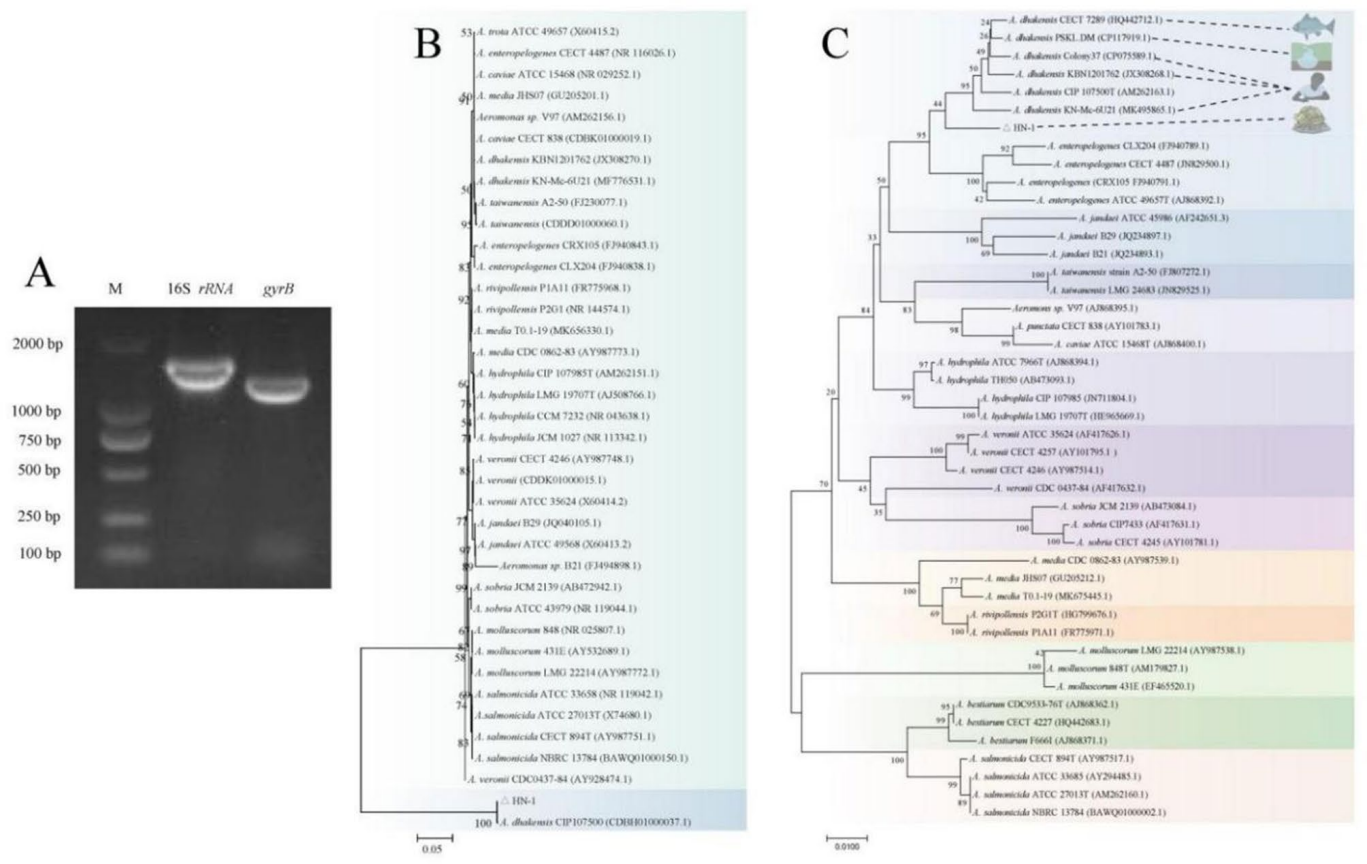
Molecular analysis of HN-1 gene amplicons. (**A**) Agarose gel electrophoresis of PCR products from bacterial isolate HN-1 for 16S *rRNA* and *gyrB*. Lane M: DL2000 DNA marker. (**B**-**C**) Unrooted neighbor-joining phylogenetic trees of the bacterial isolate HN-1 sequence (in bold) and sequences with highest identity of *Aeromonas* species, for the 16S *rRNA* gene (**B**), and *gyrB* gene (**C**). Numbers at nodes indicated bootstrap percentages obtained after 1000 replicates

The newly isolated bacterial strain HN-1 was subjected to further identification through analysis of the *16S rRNA* and *gyrB* gene sequences. The lengths of the *16S rRNA* gene and *gyrB* gene from the isolated strain HN-1 were 1446 bp (GenBank accession number: OQ380941) and 1191 bp (GenBank accession number: OQ743451), respectively (Fig. 4A). Blast analysis in NCBI revealed that the *16S rRNA* and *gyrB* gene sequences of the isolated strain HN-1 exhibited 96.65–99.73% and 95.73–98% similarity to those of *Aeromonas* strains, respectively. Two phylogenetic trees were constructed using the *16S rRNA* gene and *gyrB* gene sequences of the isolated strain HN-1 as well as the reference strains. The *16S rRNA* phylogenetic tree showed that HN-1 clustered only with *dhakensis* CIP107500 (Fig. 4B). Similarly, the *gyrB* phylogenetic tree demonstrated a closely knit cluster formed by the strain HN-1 and all *A. dhakensis* strains (Fig. 4C). Based on morphological characteristics, MALDI-TOF test, and analysis of the *16S rRNA* and *gyrB* gene sequences, the isolated bacterial strain HN-1 was identified as *A. dhakensis*.

### *A. dhakensis* HN-1 contains 7 virulence genes

**Fig. 5:**
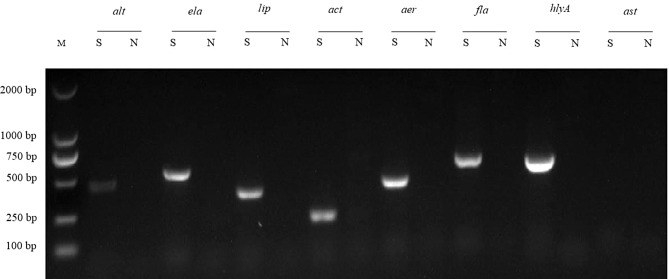
Agarose gel electrophoresis of PCR products from *A. dhakensis* HN-1 for detecting eight virulence genes. Lane M: DL2000 DNA marker. S indicates the nucleic acid samples from *A. dhakensis* HN-1. N indicates negative control samples

Eight virulence associated genes of *A. dhakensis* strain HN-1 were evaluated using PCR to determine its virulence. The results revealed that *A. dhakensis* HN-1 possesses 7 virulence genes, namely *alt* (encoding for heat-labile cytotoxic enterotoxin), *ela* (encoding for elastase), *lip* (encoding for lipase), *act* (encoding for cytotoxic enterotoxin), *aerA* (encoding for aerolysin), *fla* (encoding for polar flagella), and *hlyA* (encoding for hemolysin) (Fig. 5). However, the *ast* virulence gene was found to be absent (Fig. 5).

### *A. dhakensis* HN-1 causes pathological changes of multiple organs in mice

**Fig. 6:**
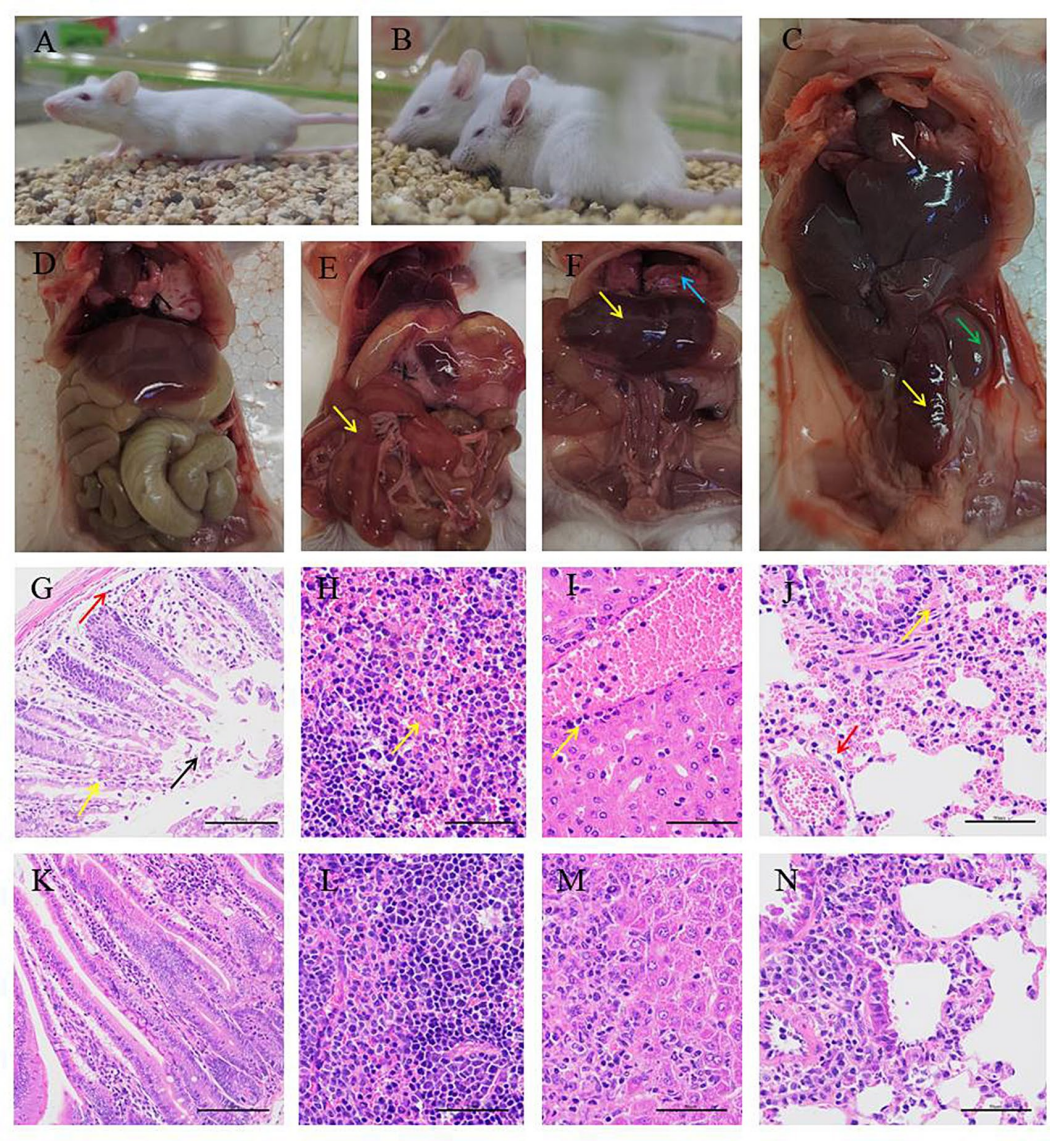
The pathological changes of *A. dhakensis* HN-1 infected mice. (**A**, **D**) No obvious clinic symptoms and visible lesions in the negative control group; (**B**) Mental malaise, lethargy and tremor in the infected groups; (**C**) Swelling and hemorrhage in the spleen (yellow arrow) and kidney (green arrow); (**E**) Oedema with acute hemorrhage in the intestine wall (yellow arrow); (**F**) Hepatic oedema (yellow arrow), severe hemorrhage in the lung (blue arrow); (**G**) Intestine mucosal epithelial cells shedding (black arrow), capillary congestion expansion (yellow arrow), mucosal layer and submucosa infiltrated with a small amount of inflammatory cell (red arrow). (**H**) Spleen red pulp congestion; (**I**) Inflammatory cell infiltration around the hepatic portal area; (**J**) Mucosal epithelial cells arranged irregularly (yellow arrow), perivascular inflammatory cell infiltration (red arrow); (**K**-**N**) PBS control

Five groups of mice (six mice per group) were inoculated intraperitoneally with the newly identified *A. dhakensis* strain, with doses ranging from 8.15 × 10^5^ CFU/mouse to 6.6 × 10^7^ CFU/mouse. Within 12 h post-inoculation, the mice exhibited symptoms such as lethargy, tremor, and tachypnea, which were similar to those observed in the deceased Aldabra giant tortoises. The mortality of the mice started 24 h post-inoculation, with all the mice in the two highest dose groups dying within two days. The mortality rates within seven days for the other three groups, inoculated with doses of 7.3 × 10^6^ CFU/mouse, 2.4 × 10^6^ CFU/mouse, and 8.15 × 10^5^ CFU/mouse, were 57%, 12.5%, and 0%, respectively. The control group showed no obvious changes, with the mice appearing active and having a good appetite (Fig. 6A, D). The LD_50_ value for the *A. dhakensis* HN-1 strain in mice was estimated to be 2.05 × 10^7^ CFU/mL (Table S2). Furthermore, the pathological changes observed in the heart, lung, liver, and spleen of the infected mice were similar to those observed in the deceased Aldabra giant tortoises (Fig. 6B-C, E-J). Additionally, the recovered isolates from the infected mice were identified as *A. dhakensis* through *16S rRNA* gene and *gyrB* gene sequence analysis.

### *A. dhakensis* HN-1 is a multidrug-resistant strain

The susceptibility of *A. dhakensis* HN-1 to 19 antibiotics was tested using disk diffusion. The results indicated that HN-1 was highly resistant to amoxicillin, penicillin, ampicillin and erythromycin. In particular, it was highly resistant to three β-lactams antibiotics, and the inhibition zone diameter was almost zero. However, it was found to be sensitive to gentamicin, amikacin, spectinomycin, kanamycin, norfloxacin, levofloxacin, doxycycline, ceftriaxone, cefoperazone, ceftazidime, and fosfomycin (Table S3).

## Discussion

To date, there have been several reports of *A. dhakensis* as a causative pathogen in various animals worldwide. These include dolphins and eels in Spain [7, 14], pacu fish in Brazil [35], oil sardine in India [36], aquaculture fish in South Korea [21], Nile tilapia in Mexico [22], striped catfish in Vietnam [15] and Malaysia [37], sea lions and farmed Siamese crocodiles in China [16, 23]. However, there have been no previous reports of *A. dhakensis* infection in tortoises. In this study, we observed that multiple organs of the Aldabra giant tortoise exhibited acute lesions such as hemorrhage and necrosis, resulting in a fatal infection caused by *A. dhakensis*. This is the first report of *A. dhakensis* infection in Aldabra giant tortoises, expanding the known infection spectrum of *A. dhakensis* which was previously limited to aquatic animals and humans. It is worth noting that *A. dhakensis* has been associated with various diseases in humans worldwide [10, 18, 26, 38–40]. Moreover, it is the most virulent *Aeromonas* species in both humans and animals [23, 24], and continues to be the predominant *Aeromonas* species among clinical isolates [23, 26, 40]. These findings highlight the clinical correlation of *A. dhakensis* and its potential to pose public health concerns. In fact, *A. dhakensis* is increasingly recognized as a zoonotic pathogen, as it has been isolated from various clinical and environmental samples worldwide [24]. Despite being known for their extraordinarily long lives, our research suggests that even Aldabra giant tortoises can be susceptible to fatal *A. dhakensis* infections. The primary diet of Aldabra giant tortoises consists of vegetation such as leaves, grasses, woody plant stems, various fruits, sedges, and herb plants [28]. Additionally, although not permitted, tourists sometimes feed Aldabra giant tortoises carrots, sweet potatoes, and other types of browse. Therefore, it is likely that the Aldabra giant tortoise was infected through the ingestion of water and food contaminated with *A. dhakensis*.

In this study, *A. dhakensis* HN-1 was found to carry 7 virulence genes, namely *alt*, *ela*, *lip*, *act*, *aerA*, *fla*, and *hlyA*. These virulence genes are considered to be the main factors influencing the pathogenicity of *Aeromonas*. Previous research on *Aeromonas* infections has shown that the virulence phenotype is a result of the cumulative effect of multiple pathogenic factors [41]. *A. dhakensis* is known to be the most virulent species within the genus *Aeromonas* due to a higher virulence gene content and hemolytic and proteolytic activities [23, 27]. The presence of polar flagella (*fla*) allows Aeromonas bacteria to move rapidly on solid surfaces and form biofilms [42, 43]. The cytotoxic enterotoxin (*act*) inhibits phagocytosis activity in host cells and causes hemolysis [44]. The *aerA* gene encodes aerolysin, which is the prototype hemolysin of the genus *Aeromonas*. It forms pores in the target cell membrane, leading to osmotic lysis [43, 45]. Additionally, A. *dhakensis* has shown higher virulence than *A. hydrophila* and *A. jandaei*, with an LD_50_ value of 8.91 × 10^5^ CFU/mL in Siamese crocodiles [23]. In this study, the LD_50_ value of *A. dhakensis* strain HN-1 was estimated to be 2.05 × 10^7^ CFU/mL for mice. These findings suggest that *A. dhakensis* HN-1 has a strong potential for infection and invasion.

Antimicrobial resistance in *A. dhakensis* has become an increasingly concerning issue in both humans and animals. Previous studies have found that most *A. dhakensis* strains, regardless of whether they were isolated from humans or animals, exhibit resistance to amoxicillin, ampicillin, and penicillin [13, 20, 25, 27, 46]. In alignment with these findings, the present study also found resistance to these antibiotics in the *A. dhakensis* HN-1 strain isolated from the Aldabra giant tortoise. Additionally, research conducted in South Korea discovered that *Aeromonas* strains found in zebrafish exhibited resistance to imipenem (65.1%) and cephalothin (25.58%) [13], which are prohibited for use in veterinary clinics. Carbapenems are deemed critically significant antimicrobial agents for humans by the World Health Organization. Recent studies have shown that clinical *A. dhakensis* isolates from humans in both Malaya and Singapore have a high resistance to carbapenems. These isolates have demonstrated resistance to imipenem (76.9%), doripenem (62.4%), and meropenem (41.9%) [23]. Interestingly, *A. dhakensis* HN-1 strain in this study is temporarily sensitive to Carbapenems, which may be related to the fact that tortoises rarely use large amounts of these antibiotics in the zoo. To further evaluate the drug resistance potential of HN-1, we conducted PCR tests on 10 common drug resistance genes, namely Tem, qnrS, IMP, AmpC, qepA, ctxM, qnrB, qnrA, parC, OXA-23, and OXA-24, for this bacterium. Our analysis revealed that this bacterium lacks the necessary resistance genes to confer resistance to most antibiotics (**Data not shown**). In the future, we will continue to track and monitor the drug resistance and resistance gene of *A. dhakensis* isolates in the zoo.

It is essential to establish a comprehensive prevention and control strategy for *A. dhakensis*, one of the most prevalent pathogenic Aeromonas species with a global distribution. This bacterium has a significant impact on the health of livestock, poultry, and aquatic animals, and has been identified as a human pathogen, posing a zoonotic threat. Additionally, drug resistance of *A. dhakensis* in farmed animals and wildlife poses a serious threat to human health. To address these concerns, several key measures should be taken based on One health framework. Firstly, early detection technology for *A. dhakensis* should be established, and collaborative efforts across multiple departments are needed to conduct epidemiological investigations of the disease in humans, animals, and the environment. Secondly, research into the mechanism of infection, pathogenicity, and drug resistance of the disease should be prioritized, with a focus on the development of broad-spectrum effective vaccines and clinical therapy drugs. Finally, the rational use of antibiotics in animals should be enforced, with regular monitoring of antimicrobial resistance and resistance genes at designated points. These measures are critical for the effective management of *A. dhakensis* and for safeguarding human and animal health.

## Conclusion

*A. dhakensis* was isolated from Aldabra giant tortoise for the first time, thereby expanding its host spectrum. The *A. dhakensis* HN-1 strain was found to possess 7 virulence genes, indicating its strong zoonotic potential. In light of these findings, further studies are warranted to investigate the epidemiological characteristics of *A. dhakensis*, including active surveillance, pathogenesis, and antibiotic sensitivity. This information will be crucial in guiding the management of future human and animal infections.

### Electronic supplementary material

Below is the link to the electronic supplementary material.

Supplementary Material 1: Table S1. Sequence of oligonucleotides and PCR conditions used in the study

## Data Availability

No datasets were generated or analysed during the current study.
