# Supplementary material for: Exploring Aeromonas dhakensis in Aldabra giant tortoises: a debut report and genetic characterization
Source: BMC Microbiol. 2024 Mar 7;24:76. doi: 10.1186/s12866-024-03203-w (PMC10921707; doi:10.1186/s12866-024-03203-w)
Supplement: Supplementary file 1 — Supplementary Material 1: Table S1. Sequence of oligonucleotides and PCR conditions used in the study [file 12866_2024_3203_MOESM1_ESM.docx]

Table S1. Sequence of oligonucleotides and PCR conditions used in the study

| Gene | Primer | Primer Sequence (5’-3’) | Length (bp) | References |
| --- | --- | --- | --- | --- |
| *Aer* | *aer*-F | CCTATGGCCTGAGCGAGAAG | 431 | Igbinosa and  Okoh, 2013 |
|  | *aer*-R | CCAGTTCCAGTCCCACCACT |  |  |
| *HlyA* | *hlya*-F | GGCCGGTGGCCCGAAGATACGGG | 597 |  |
|  | *hlya*-R | GGCGGCGCCGGACGAGACGGG |  |  |
| *Fla* | *fla*-F | TCCAACCGTYTGACCTC | 608 |  |
|  | *fla*-R | GMYTGGTTGCGRATGGT |  |  |
| *Lip* | *lip*-F | ATCTTCTCCGACTGGTTCGG | 382 | Sen and Rogers,  2004 |
|  | *lip*-R | CCGTGCCAGGACTGGGTCTT |  |  |
| *Alt* | alt-F | TGACCCAGTCCTGGCACGGC | 442 |  |
|  | alt-R | GGTGATCGATCACCACCAGC |  |  |
| *Ast* | *ast*-F | TCTCCATGCTTCCCTTCCACT | 331 |  |
|  | *ast*-R | GTGTAGGGATTGAAGAAGCCG |  |  |
| *Act* | *act*-F | AGAAGGTGACCACCAAGAACA | 232 |  |
|  | *act*-R | AACTGACATCGGCCTTGAACTC |  |  |
| *Ela* | *ela*-F | ACACGGTCAAGGAGATCAAC | 513 |  |
|  | *ela*-R | CGCTGGTGTTGGCCAGCAGG |  |  |

Table S2. The half-maximal lethal dose (LD_50_) assay of *A. dhakensis* HN-1

| Group | Dose（CFU/mouse）  (0.3 mL/mouse) | Death number  (6 mice per group) | Mortality rates |  |
| --- | --- | --- | --- | --- |
|  |  |  |  | LD_50_  (CFU/mL) |
| 1 | 6.6×10^7^ | 6 | 100% | 2.05×10^7^ |
| 2 | 2.2×10^7^ | 6 | 100% |  |
| 3 | 7.3×10^6^ | 3 | 57% |  |
| 4 | 2.4×10^6^ | 1 | 12.5% |  |
| 5 | 8.15×10^5^ | 0 | 0% |  |

Table S3. The antibiotics susceptibility of *A. dhakensis* HN-1

| Category of antibiotics | Antimicrobial agent | Disk content（μg） | Standard diameter of inhibited zone/mm | | | Diameter of inhibited zone/mm | Susceptibility |
| --- | --- | --- | --- | --- | --- | --- | --- |
|  |  |  | Resistance | Intermediate | Susceptible |  |  |
| Rifampicin | Rifampicin | 5 | ≤16 | 17-19 | ≥20 | 12.5 | R |
| Aminoglycosides | Gentamicin | 10 | ≤12 | 13-14 | ≥15 | 15.35 | S |
|  | Amikacin | 30 | ≤14 | 15-16 | ≥17 | 18.3 | S |
|  | Spectinomycin | 100 | ≤14 | 15-17 | ≥18 | 19.6 | S |
|  | Kanamycin | 30 | ≤13 | 14-17 | ≥18 | 20.35 | S |
| Quinolones | Norfloxacin | 10 | ≤12 | 13-16 | ≥17 | 26.5 | S |
|  | Levofloxacin | 5 | ≤13 | 14-16 | ≥17 | 26 | S |
|  | Ciprofloxacin | 5 | ≤15 | 16-20 | ≥21 | 26 | S |
| Tetracyclines | Doxycycline | 30 | ≤11 | 12-14 | ≥15 | 20.75 | S |
| Sulfonamides | Trimethoprim /sulfamethoxazole | 23.75/1.25 | ≤10 | 11-16 | ≥17 | 16.3 | I |
| β-lactams | Amoxicillin | 20 | ≤13 | 14-17 | ≥18 | 0 | R |
|  | Penicillin | 10 U | ≤11 | 12-14 | ≥15 | 0 | R |
|  | Ampicillin | 10 | ≤13 | 14-16 | ≥17 | 0 | R |
|  | Ceftriaxone | 30 | ≤19 | 20-22 | ≥23 | 24.3 | S |
|  | Cefoperazone | 75 | ≤15 | 16-20 | ≥21 | 26 | S |
|  | Ceftazidime | 30 | ≤17 | 18-20 | ≥21 | 22.35 | S |
| Macrolides | Erythromycin | 15 | ≤13 | 14-22 | ≥23 | 10.1 | R |
| Fosfomycin | Fosfomycin | 200 | ≤12 | 13-15 | ≥16 | 39.55 | S |

Notes: Susceptible(S); Intermediate(I); Resistance(R).
